# Seroconversion Is Misleading as a Test for HSV-2 Infection in Prophylactic Genital Herpes Vaccine Trials: Results of Vaccine Studies in Guinea Pigs

**DOI:** 10.3390/v17060773

**Published:** 2025-05-29

**Authors:** Valerie Bromberg, Lauren M. Hook, John M. Lubinski, Zauraiz Syeda, Kevin P. Egan, Gary H. Cohen, Sita Awasthi, Harvey M. Friedman

**Affiliations:** 1 Infectious Disease Division, Department of Medicine, Perelman School of Medicine, University of Pennsylvania, One UCity, 25 N 38th St, Room 4025, Philadelphia, PA 19104-5956, USA; valerie.bromberg@pennmedicine.upenn.edu (V.B.); lhook@pennmedicine.upenn.edu (L.M.H.); john.lubinski@pennmedicine.upenn.edu (J.M.L.); zauraizs@gmail.com (Z.S.); kevinpe@pennmedicine.upenn.edu (K.P.E.); sawasthi@pennmedicine.upenn.edu (S.A.); 2 Department of Basic and Translational Sciences, School of Dental Medicine, University of Pennsylvania, Philadelphia, PA 19104, USA; ghc@upenn.edu

**Keywords:** seroconversion, gG2 ELISA, Western blot, HSV-2 vaccine, genital infection, genital lesions, genital shedding of HSV-2 DNA

## Abstract

Seroconversion is defined as a four-fold or greater rise in antibody titers. This assay is used in human prophylactic vaccine trials to confirm HSV as the cause of genital lesions and detect subclinical latent infection. We evaluated the accuracy of seroconversion in detecting infection using a guinea pig model of genital infection. Not all animals intravaginally inoculated with HSV-2 become infected, particularly if vaccinated; therefore, we need to establish criteria to determine whether an animal is infected. Our primary analysis involved considering animals to be infected if they had any of the following: (a) genital lesions; (b) HSV-2 DNA in vaginal secretions four or more weeks after HSV-2 inoculation as a marker of reactivation from latency; or (c) HSV-2 DNA in dorsal root ganglia, the site of latency. In the second analysis, we considered animals to be infected if they had positive virus cultures from vaginal swabs obtained on day two or four post HSV-2 inoculation. In the third analysis, we considered animals to be infected if they had any condition included in the first two analyses. We collected sera prior to HSV-2 inoculation and two months later and tested the first 57 animals for seroconversion using Western blotting and gG2 IgG ELISA. The results were concordant in 54 of 57 animals (95%), and when discordant, the gG2 ELISA matched infection results as defined by the primary analysis. The remaining animals were evaluated by gG2 IgG ELISA only. A total of 43 animals were inoculated with HSV-2 but not vaccinated (*No vaccine* group), and 224 were vaccinated with glycoprotein or mRNA vaccines prior to HSV-2 inoculation (*Vaccine* group). In the *No vaccine* group, we detected no false positives (seroconversion without infection) but 24% to 29% false negatives (no seroconversion despite infection) depending on the criteria used to define infection. In the *Vaccine* group, we detected 8% to 22% false positives and 31% to 37% false negatives. The accuracy of seroconversion was 74% to 79% in the *No vaccine* group and 71% to 76% in the *Vaccine* group. These results raise concerns about using seroconversion as a diagnostic test in human vaccine trials. Alternate approaches, such as subject home swabbing for HSV DNA, should be considered as a possible replacement.

## 1. Introduction

An accurate diagnosis of genital herpes infection in prophylactic vaccine trials is required for reliable assessments of vaccine efficacy. Three large prophylactic vaccine efficacy trials have been reported [1,2,3]. The primary endpoint for two of these vaccine trials was genital lesions, while the third included both genital lesions and latent infection [1,2,3]. In clinical trials and general practice, HSV genital lesions can be difficult to distinguish from syphilis, chancroid, and non-infectious causes [4,5,6]. In one study involving 74 subjects diagnosed with genital herpes, 14 were misdiagnosed (19%) [7]. Subjects in the three published vaccine efficacy trials with genital lesions were asked to contact the study site and come to the clinic for an examination and laboratory tests to confirm the diagnosis [1,2,3]. The preferred confirmatory test was either a swab for HSV DNA by PCR or virus culture; however, these tests were not performed if the lesions developed after clinic hours or on weekends or if lesions healed before the visit. In these circumstances, seroconversion, defined as a four-fold or greater rise in antibody titers against antigens not induced by the vaccine, was used to confirm the diagnosis of genital herpes [1,2,3]. Seroconversion was also used to diagnose subclinical (latent) infection in subjects with no genital lesions [1,2,3].

Assays used to detect seroconversion included Western blots and IgG ELISA [1,2,3]. Despite their use in each of the phase 3 genital herpes vaccine trials, the accuracy of seroconversion has not been established. Reasons to be concerned about its accuracy include the possibility that some vaccinated individuals may seroconvert despite infection being so mild that genital lesions and latency do not develop. These individuals would be classified as vaccine failures despite having clinically insignificant infection. Other individuals may fail to seroconvert despite developing genital lesions or latent infection and would be considered uninfected and having vaccine successes despite the presence of infection.

We assessed the accuracy of seroconversion for confirming the diagnosis of HSV-2 genital lesions or detecting latent infection in guinea pigs that were either immunized or not immunized prior to intravaginal HSV-2 inoculation [8]. Other investigators have used seroconversion in animal models to demonstrate antibody responses to HSV-2 antigens not included in the vaccine but have not assessed the accuracy of seroconversion for detecting HSV infection [9,10]. Over a 10-year period, we immunized guinea pigs with various experimental HSV-2 vaccines prior to intravaginal inoculation with HSV-2, while other animals were not immunized, enabling us to assess seroconversion in vaccinated and unvaccinated control animals. We evaluated paired sera for seroconversion using a sample obtained prior to infection and another approximately two months later. We calculated the accuracy of seroconversion based on a variety of criteria used to determine whether an animal was infected, including genital lesions, asymptomatic subclinical infection based on daily vaginal swabs for HSV-2 DNA obtained over a three-week period starting on day 29 post inoculation, latent infection determined by detecting HSV-2 DNA in dorsal root ganglia (DRGs), and virus titers in vaginal swabs obtained on days two and four post inoculation. Seroconversion assays had high false negative error rates in both vaccinated and unvaccinated animals, while high false positive rates were only detected in vaccinated animals, raising concerns about using this assay to confirm genital infection in prophylactic vaccine trials.

## 2. Methods

Vaginal swab virus titers, genital lesions, vaginal shedding of HSV-2 DNA, and HSV-2 DNA in DRGs were assessed. The following methods were used. 

UV inactivation of HSV-2: Virus was placed in a tissue culture dish immediately adjacent to the UV bulb in a laminar airflow hood for 30 min, and the dish was gently rocked every 10 min. Virus inactivation was confirmed by plaque assay on Vero cells [11].

Vaginal inoculation and day two and day four vaginal virus titers: Intravaginal inoculations were performed using replication competent HSV-2 strain MS. Vaginal swabs for virus titers were obtained on days two and four post inoculation. The vaginal swabs were placed in 1 mL Dulbecco’s modified Eagles medium (DMEM) with 5% fetal bovine serum and 25 μg/mL vancomycin. Virus titer was determined by plaque assay on Vero cells using 150 μL of undiluted swab media and serial 10-fold dilutions. The assay had a lower limit of detection 6.7 PFU/mL [11].

Guinea pigs and scoring for genital lesions: Female Hartley strain guinea pigs from Charles River Laboratories weighing 250 to 350 g were used. Animals were scored for genital lesions after inoculation with replication competent HSV-2. We defined a lesion as an area on the external genitalia that was red and raised with a white or fluid-filled center. The daily scores assigned to each animal were based on a consensus of two investigators that were blinded to the group, and both had to agree on whether a lesion was present [12].

Vaginal shedding of HSV-2 DNA during the recurrent phase of infection: Vaginal swabs for HSV-2 DNA were performed for 20–21 days, generally on days 29–49 post inoculation. Vaginal swabs were placed in 1 mL of DMEM (containing HEPES, L-glutamine, and antibiotics) and 5% fetal bovine serum and frozen at −80 °C. Once thawed, 200 μL was used to purify DNA (QiaCube HT, QIAGEN, Germantown. MD, USA.), and 5 μL of purified DNA was amplified (Roche LightCycler 96, Temecula, CA, USA). HSV-2 U_S_9 primers and probes were used for detection and DNA copy number quantitation [13]. Samples with less than 1 copy of HSV-2 DNA by 40 cycles were considered negative, while positive samples were repeated and confirmed in duplicate. The assay limit of detection was 200 copies of HSV-2 DNA/mL [14].

Detecting HSV-2 DNA in DRG: Guinea pig DRGs were placed in 1 mL of DMEM containing 5% fetal bovine serum and frozen at −80 °C. After thawing, the media were removed and lysis buffer (Qiagen) was added to DRGs overnight at 56 °C. An amount of 200 μL was processed for DNA purification, and 5 μL of purified DNA was amplified in duplicate (Roche LightCycler 96) using U_S_9 primers and probes for detection and quantitation. HSV-2 copy number was calculated as log_10_ DNA copies per 10^6^ glyceraldehyde-3-phosphate dehydrogenase (GAPDH) genes [12].

### Seroconversion Assays

Serum was obtained from the left hind limb saphenous vein. For unvaccinated animals, the pre-inoculation (acute) serum was obtained just before virus inoculation, and the second serum at least 2 months later. For vaccinated animals, we collected the first serum sample at least one month after final immunization and prior to HSV-2 infection, while the second serum sample was obtained approximately 2 months later. For experiments using UV-inactivated virus, the first serum sample was obtained prior to UV virus inoculation, and the second serum sample was collected after 1.3 to 3 months. We defined a false positive result as seroconversion in an animal that did not have infection as determined using the Lesions/Latency, Virus Replication, or Combined criteria. A false negative result was an animal with infection that failed to seroconvert.

ELISA: Each animal included in the study had paired acute and convalescent sera tested using IgG ELISA for seroconversion to HSV-2 gG2 [15]. Ninety-six-well plates were coated with 100 ng gG2/well (Abcam, Waltham, MA, USA) and blocked with 5% nonfat milk. Serial serum dilutions with ratios ranging from 1:50 to 1:6400 were added to gG2-coated wells, followed by HRP-conjugated anti-guinea pig IgG. Optical density (OD) was measured at 405 nm after 15–30 min. The endpoint titer was considered the highest dilution of serum with an OD reading at least 2-fold higher than the average OD of 8 wells that contained all reagents except guinea pig serum. Seroconversion was defined as ≥4-fold rise in titer comparing acute and convalescent sera. Any sera pairs showing a 2-fold rise were repeated. Upon repetition, all showed either no rise or ≥4-fold rise.

Western blotting: A low-passage clinical isolate, namely HSV-2 strain 2.12, was purified and used at 5 × 10^5^ PFU combined with PBS and 4× protein loading buffer to a final volume of 120 μL and added to a broad single well of a 4–12% SDS PAGE gel (Invitrogen, Carlsbad, CA, USA). Comb teeth were used to separate the first and last lanes from the broad lane containing HSV-2. The first lane was used to run molecular weight markers, while the last lane contained gC2, gD2, and gE2 proteins at a final concentration of 10 ng each to indicate positions of vaccine antigens. The gel contents were transferred to a PVDF membrane, and the broad HSV-2 lanes were cut into individual strips, probed with acute or convalescent guinea pig sera at a 1:100 dilution. The lanes containing gC2, gD2, and gE2 proteins were probed with rabbit antibodies UP2151 to gC2 and R265 to gE2 and with mouse antibody ID3 to gD2. HRP-conjugated anti-guinea (1:25,000), anti-rabbit, or anti-mouse (1:2000) IgG was added to detect HSV-2 bands. ECL reagent was used to develop the blot. Seroconversion was defined by the appearance of HSV-2 bands in blots incubated with convalescent serum that were not present in the acute serum and that were not produced by vaccine antigens.

## 3. Statistics

*p* values for differences in seroconversion rates between groups were calculated using the two-tailed Fisher’s exact test. The two-tailed Mann–Whitney test was used to detect differences between peak vaginal virus titers on days 2 and 4. *p* values < 0.05 were considered significant.

### Institutional Animal Care and Use Study Approval

Animal studies were conducted under protocol #805187 that was approved by the Institutional Animal Care and Use Committee of the University of Pennsylvania. The protocol abides by the “Guide for the Care and Use of Laboratory Animals” written by the Institute for Laboratory Animals Research Guidelines. Guinea pigs were euthanized at the end of the experiment or if they reached a humane endpoint. Criteria for humane euthanasia included necrotic genital lesions, four consecutive days of urinary retention, four consecutive days of bloody urine, unilateral or bilateral hind limb paralysis, or 20% or more loss of pre-inoculation weight. Euthanasia was accomplished using Euthasol Solution C3N (Covetrus, Portland, ME, USA) in an amount of 100–400 mg/kg administered intraperitoneally by injection. Animals were assessed for appropriate anesthetic depth and terminally bled via cardiac puncture after no response to pedal reflex was noted. Additional 1/4 to 1/2 dose of Euthasol was administered if a pedal reflex was not ablated. Cessation of respiration and no heartbeat were used to verify euthanasia.

## 4. Results

### 4.1. Guinea Pig Studies

**Figure 1:**
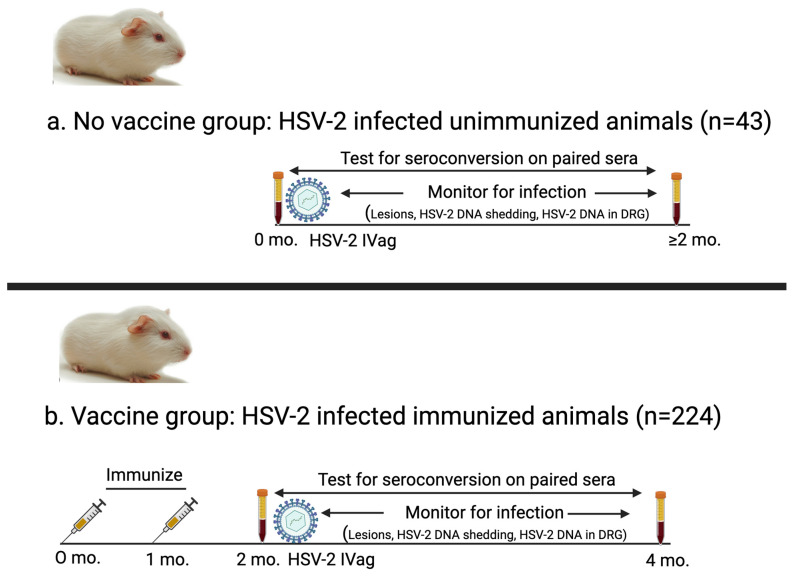
Schematic of experimental design for animals in *No vaccine* and *Vaccine* groups. (**a**) *No vaccine* group: Serum was obtained immediately prior to HSV-2 intravaginal inoculation and again two months later. Animals in this group were not immunized. (**b**) *Vaccine* group: Guinea pigs were immunized two or three times (two shown here) with protein or mRNA vaccines, and serum (acute sample) was obtained one month after final immunization. Animals were inoculated intravaginally with HSV-2 after obtaining first serum sample and then evaluated for infection. Second convalescent serum sample was obtained two months after first serum sample.

We performed nine studies in female Harley strain guinea pigs over a 10-year period to evaluate experimental vaccines for genital herpes. We analyzed serum and infection data collected from these studies to assess the accuracy of seroconversion to diagnose infection. Paired serum samples from 267 guinea pigs were separated into two groups (Figure 1). (i) The first group was the *No vaccine* group (*n* = 43) that consisted of control animals in therapeutic vaccine trials that were inoculated intravaginally with HSV-2 and not immunized [16]. We included this group to evaluate seroconversion in unvaccinated animals as a comparator for seroconversion in vaccinated animals. (ii) The second group was the *Vaccine* group (*n* = 224) that included animals vaccinated with an HSV-2 vaccine containing one of the following: (a) gD2 protein expressed in baculovirus and administered with CpG/alum; (b) gC2, gD2, and gE2 proteins expressed in baculovirus given with CpG/alum (trivalent protein vaccine); or (c) gC2, gD2, and gE2 nucleoside-modified mRNA encapsulated in lipid nanoparticles (LNPs) (trivalent mRNA vaccine) [17,18]. Animals that received baculovirus protein antigens were immunized three times with 10 μg of each protein, while animals that received mRNA vaccines were immunized two or three times using 5, 10, or 20 μg of each mRNA (Table 1) [12].

### 4.2. Assays Used to Diagnose Infection

We used the female guinea pig genital infection model to determine the accuracy of seroconversion for diagnosing infection. Not all guinea pigs inoculated intravaginally with HSV-2 develop infection, particularly those that are protected by a vaccine. Therefore, we need to establish criteria for diagnosing infection that could serve as the gold standard when assessing the diagnostic accuracy of seroconversion. We used three criteria for diagnosing infection. The primary analysis, referred to as the Lesions/Latency method, required animals to have at least one of the following: (i) one or more days with genital lesions; (ii) one or more days with a positive result according to PCR for HSV-2 DNA in vaginal secretions detected during the recurrent phase of infection (after day 28); and (iii) HSV-2 DNA detected by PCR in dorsal root ganglia (the site of latency) at the end of the experiment. We had information for all three criteria for 267 animals. The second analysis for diagnosing infection, referred to as the Virus Replication method, measured HSV-2 virus titers in vaginal swab samples obtained on days two and four post inoculation. We collected day two and four vaginal swabs from 232 of the 267 (87%) animals and included these animals in the second analysis. The third analysis included any condition described in the first two analyses. Animals were considered infected if they met any of the criteria listed for the Lesions/Latency or Virus Replication method.

### 4.3. Assays Used to Detect Seroconversion

No commercial antibody assays are available for diagnosing HSV-2 infection in guinea pigs. In humans, antibody assays are not recommended to screen single serum samples for HSV-2 infection in asymptomatic individuals because commercially available tests have many false positive results when distinguishing HSV-2 from HSV-1 antibodies [19,20]. Western blots are more accurate for screening single serum samples, but the test is not commercially available [21]. Strong concordance between Western blots and gG immunoblots was noted for diagnosing HSV-2 but not HSV-1 infection using single serum samples obtained at least 21 days after culture-proven genital herpes [22]. In our study, distinguishing HSV-1 from HSV-2 was not necessary because animals were only inoculated with HSV-2, and all animals were HSV-naïve prior to infection. In addition, we had paired sera available where the first sample was obtained prior to HSV-2 inoculation and the second sample was obtained two months later. The availability of paired sera enabled us to evaluate changes in antibody titers by comparing the two samples rather than relying on a single serum sample for diagnosis.

**Figure 2:**
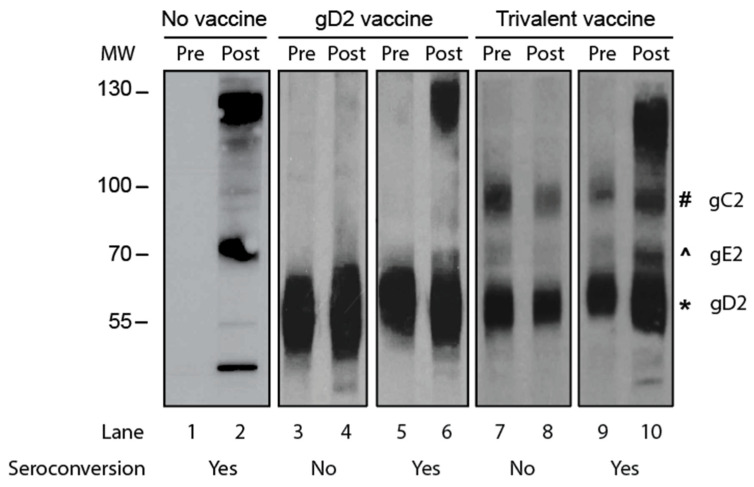
A Western blot to detect seroconversion. Purified HSV-2 virions were the source of the proteins. Lanes 1 and 2: The animal was inoculated intravaginally with HSV-2 but not vaccinated. Lanes 3–6: Animals were immunized with the gD2 subunit protein vaccine and then inoculated intravaginally with HSV-2. Lanes 7–10: Animals were immunized with a vaccine containing gC2, gD2, and gE2 subunit proteins and then inoculated intravaginally with HSV-2. MW, molecular weight markers; Pre, acute sera taken prior to HSV-2 inoculation; Post, convalescent sera obtained 2 months after HSV-2 inoculation. The symbols next to lane 10 show the positions of the gC2, gE2, and gD2 proteins.

We compared the accuracy of gG2 IgG ELISA with Western blotting for seroconversion. The two sera samples from the animal were tested on the same ELISA plate or same Western blot gel. The criterion for seroconversion for the ELISA was a four-fold or greater rise in gG2 antibody titers when comparing the first and second sera samples. The criterion for Western blotting was the presence of antibodies to HSV-2 antigens in the second sample that were not present in the first sample, and the new antibodies were not responsive to immunogens in the vaccine. Representative Western blots are shown in Figure 2 using paired sera samples obtained from an infected animal that was not vaccinated (Figure 2; lanes 1 and 2) or sera from animals that were immunized with a gD2 protein vaccine (Figure 2; lanes 3–6) or a vaccine containing gC2, gD2, and gE2 trivalent proteins (Figure 2; lanes 7–10). Our interpretation of the results is shown below the blot in Figure 2.

The first 57 animals were assessed by gG2 IgG ELISA and a Western blot. The analysis included 8 animals in the *No vaccine* group and 49 animals in the *Vaccine* group. Fifty-four of fifty-seven (95%) results were concordant when comparing gG2 IgG ELISA and the Western blot. Three results in the *Vaccine* group were discordant, including one where the gG2 assay was negative and two where the gG2 assay was positive for seroconversion. In all three animals, the gG2 result was considered correct based on the Lesions/Latency definition of infection. Therefore, we used gG2 ELISA to evaluate the remaining paired sera samples for seroconversion.

### 4.4. Accuracy of Seroconversion to Detect HSV-2 Genital Infection

We evaluated the accuracy of seroconversion for diagnosing infection in three separate analyses that used different criteria for infection, namely the Lesions/Latency, Virus Replication, and Combined methods. We considered the Lesions/Latency method to be the most clinically relevant because the criteria measure the most important outcomes, genital lesions and latency. We used Virus Replication because of the possibility that virus replication may trigger seroconversion even without causing lesions or latency, particularly in vaccinated animals. We used the Combined criteria to have the broadest possible definition of infection.

### 4.5. Using Lesions/Latency to Diagnose Infection

Regarding lesions, guinea pigs were inoculated intravaginally with HSV-2 and were evaluated daily for the first two weeks for genital lesions on the external genitalia and then scored for genital lesions five days per week until the end of the experiment. Acute genital lesions are easy to recognize because they look typical of HSV lesions in humans in that they are red and raised with fluid-filled centers (vesicles). Recurrent genital lesions (after day 28) tend to be smaller and are more difficult to recognize. We tried to minimize errors in scoring for genital lesions by including uninfected animals and having two experienced observers blinded to the groups [16]. The investigators agreed that a lesion was present before scoring the animal positive for genital herpes. Our error rate for assigning lesions to an uninfected animal was extremely low, namely 1 in 500 (0.2%) observation days, providing high assurance that we seldom misidentified lesions.

Regarding the detection of latent infection, some animals develop latent infection without genital lesions. We detected latent infection by screening for vaginal shedding of HSV-2 DNA in genital secretions for at least 20 days beginning on day 29 post inoculation and by evaluating for HSV-2 DNA in DRG at the end of the experiment. HSV-2 DNA in vaginal secretions and DRG have some uncertainty as markers of infection because of possible contamination during swabbing in the animal colony or when processing samples in the laboratory. To minimize errors, we included uninfected animals and uninfected swab media as controls and repeated assays if the controls indicated a problem that day.

We assessed the performance of each of the markers of infection, namely genital lesions, HSV-2 DNA in vaginal secretion, and HSV-2 DNA in DRG (Table 2). Genital lesions performed best in the *No vaccine* group, while vaginal shedding of HSV-2 DNA was the most sensitive assay in the *Vaccine* group. However, no assay was sensitive enough to be used as the only diagnostic test; therefore, we used all three. We considered an animal to be infected if one or more assays were positive and considered an animal uninfected if all three assays were negative.

### 4.6. Seroconversion Accuracy Using Lesions/Latency Criteria to Diagnose Infection

**Figure 3:**
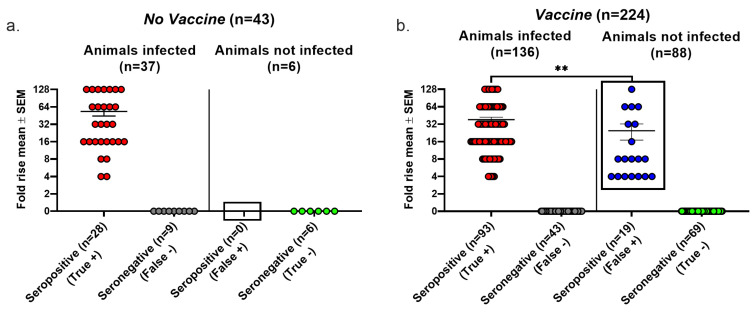
Seroconversion performance using the Lesions/Latency definition to diagnose infection. Animals were considered infected if they had at least one of the following: genital lesions, vaginal shedding of HSV-2 DNA beyond 28 days post inoculation, or HSV-2 DNA detected in DRGs. The fold rise is based on an increase in gG2 IgG titers when comparing acute and convalescent sera. A 4-fold or greater rise in titers indicates seroconversion. True positive, seroconversion in an animal with infection; false negative, no seroconversion in an infected animal; false positive, seroconversion in an uninfected animal; true negative, no seroconversion in an uninfected animal. (**a**) *No vaccine* group. (**b**) *Vaccine* group. ** *p* < 0.01.

We evaluated the false positive results, false negative results, and overall accuracy of seroconversion using the Lesions/Latency criteria to define infection. In the *No vaccine* group, 9 of 37 infected animals failed to seroconvert, producing a false negative rate of 24% (Figure 3a). We detected no false positive seroconversions in this group because no uninfected animal was seropositive, yielding a false positive rate of 0/6 (0%) (Figure 3a). In the *Vaccine* group, 43 of 136 infected animals failed to seroconvert, resulting in a false negative rate of 32%, while 19 of 88 animals that seroconverted were not infected, producing a false positive rate of 22%. The false negative rates were high in both the *No vaccine* and *Vaccine* groups, while the false positive rate was high in the *Vaccine* group, but there were no false positive results in the *No vaccine* group (compare boxed areas in Figure 3a,b) (*p* = 0.0137, two-tailed Fisher’s exact test). The true seropositive animals in the *Vaccine* group had significantly greater rises in gG2 antibody titers than the false seropositive animals (*p* < 0.01); however, 12 animals had antibody titer rises of 8- to 128-fold despite no evidence of infection. We calculated the overall accuracy of seroconversion as 79% in the *No Vaccine* group and 72% in the *Vaccine* group as follows: [(true positive + true negative)/total number of animals tested] × 100%. The high false positive and false negative rates in the *Vaccine* group and the overall accuracy of both groups are concerning.

### 4.7. Seroconversion Accuracy Using Virus Replication to Diagnose Infection

We considered the possibility that Virus Replication in the female genital tract may trigger seroconversion despite the animal having no lesions or latent infection. Therefore, we evaluated the accuracy of seroconversion using Virus Replication as the gold standard for infection. We first determined whether seroconversion requires Virus Replication. We inoculated guinea pigs intravaginally with UV-inactivated virus that contained 1–2 × 10^6^ plaque forming units of HSV-2 prior to UV inactivation. None of the eight animals in the *No vaccine* group and none of the ten animals in the *Vaccine* group had a positive virus culture on day two or four, no animal developed infection, and importantly, no animal seroconverted (Table 3). Therefore, seroconversion did not occur after intravaginal inoculation in the absence of HSV-2 replication.

We next assessed the false positive and false negative rates of seroconversion when Virus Replication was used to define infection. We collected vaginal swabs on days two and four post inoculation from 39 of 43 (91%) animals in the *No vaccine* group and 193 of 224 (86%) animals in the *Vaccine* group. In the *No vaccine* group, 6 animals had negative titers on both days, 31 animals had positive titers on both days, and 2 animals were positive on only one day. In the *Vaccine* group, 72 animals had negative titers on both days, 59 had positive cultures on both days, and 62 had positive titers on one day only. In total, 64 animals had positive titers on one day only. A total of 2 of 64 (3%) animals were positive only on day four, while 62 of 64 (97%) animals were positive only on day two, raising the possibility that the positive titer on day two may represent residual virus from the inoculum. However, 60/193 (31%) animals in the *Vaccine* group had cultures that were positive only on day two compared to 2/39 (5%) in the *No vaccine* group (*p* = 0.0005 according to two-tailed Fisher’s exact test). This disproportionate distribution in the *Vaccine* group of positive cultures on day two only supports that vaccine immunity, rather than residual virus, accounted for most animals with positive virus titers on day 2 only. Therefore, we included animals with a single positive titer on either day or positive titers on both days in our analysis.

**Figure 4:**
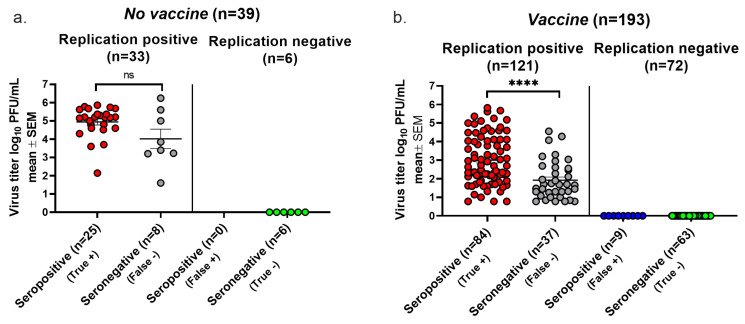
Seroconversion using Virus Replication to diagnose infection. Vaginal swabs were obtained on days two and four post infection. Animals were considered infected if virus cultures were positive on one or both days. True positive, seroconversion in an infected animal. False negative, no seroconversion in an infected animal. False positive, seroconversion in an uninfected animal. True negative, no seroconversion in an uninfected animal. (**a**) Virus titers in the *No vaccine* group. (**b**) Virus titers in the *Vaccine* group. The lower limit for detection of Virus Replication was 6.7 PFU. **** *p* < 0.0001; ns, not significant.

We plotted the highest virus titer values when both days were positive, the single-day titer when only one day was positive, and a titer of zero if neither day was positive. In the *No vaccine* group, 25 animals with positive cultures were seropositive, while 8 were seronegative, establishing a false negative rate for seroconversion of 8/33 (24%) (Figure 4a). None of the six animals with negative cultures were seropositive, yielding a false positive rate of 0/6 (0%) (Figure 4a). The replication-positive animals in the *No vaccine* group had virus titers that were not significantly different when comparing seropositive with seronegative animals, indicating that despite there being high virus titers, some animals failed to seroconvert. In the *Vaccine* group, 37 of 121 animals with positive virus cultures failed to convert, yielding a false negative rate of 31% (Figure 4b). A total of 9 of 72 animals with negative virus cultures seroconverted, resulting in a false positive seroconversion rate of 13%. Although virus titers were significantly lower in the seronegative than seropositive animals, many animals with high virus titers failed to seroconvert. The overall accuracy of seroconversion was 79% in the *No Vaccine* group and 76% in the *Vaccine* group, results that are comparable to those using the Lesions/Latency criteria to define infection.

## 5. Combined Criteria

**Figure 5:**
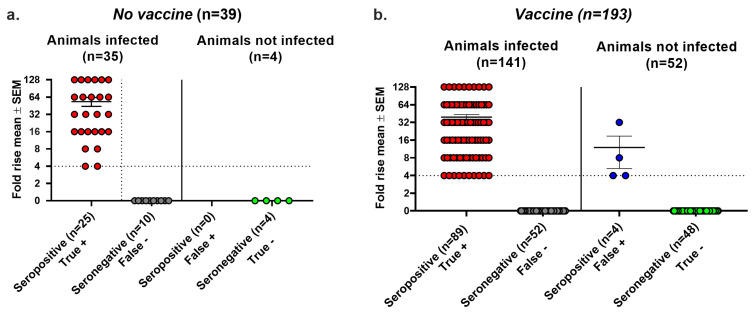
Combined criteria using Lesions/Latency and Virus Replication to define infection. Animals were considered infected if they had at least one of the following: genital lesions, vaginal shedding of HSV-2 DNA beyond 28 days post inoculation, HSV-2 DNA detected in DRG, or positive vaginal virus cultures on days 2 or 4 post inoculation. Fold rise is based on an increase in gG2 IgG titers when comparing acute with convalescent sera, with titers with ≥4-fold rise indicating seroconversion. True positive, seroconversion in an animal with infection. False negative, no seroconversion in an infected animal. False positive, seroconversion in an uninfected animal. True negative, no seroconversion in an uninfected animal. (**a**) *No vaccine* group. (**b**) *Vaccine* group.

We performed a third analysis of seroconversion accuracy using the Combined criteria of infection. Animals with at least one of the following were considered infected: (i) genital lesions; (ii) HSV-2 DNA detected in vaginal swabs during the recurrent phase of infection; (iii) HSV-2 DNA in DRG; or (iv) positive virus cultures on days two or four. Using these criteria, the *No vaccine* group had false negative results in 10 of 35 (29%) animals, false positive results in 0 of 4 (0%) animals, and an overall accuracy of 29/39 (74%) (Figure 5a). In the *Vaccine* group, we noted false negative results in 52 of 141 (37%) animals and false positive results in 4 of 52 (8%) animals, yielding an overall accuracy of 137/193 (71%) (Figure 5b). The impact of the Combined criteria was to increase false negative seroconversion rates, decrease false positive seroconversion rates, and overall, to decrease the accuracy of seroconversion. We conclude that seroconversion was inaccurate for the *No vaccine* and *Vaccine* groups using any of the three methods to define infection, namely Lesions/Latency, Virus Replication, and the Combined criteria (Table 4).

### 5.1. The Accuracy of Seroconversion as a Confirmatory Test for Genital Lesions or Subclinical (Latent) Infection

**Figure 6:**
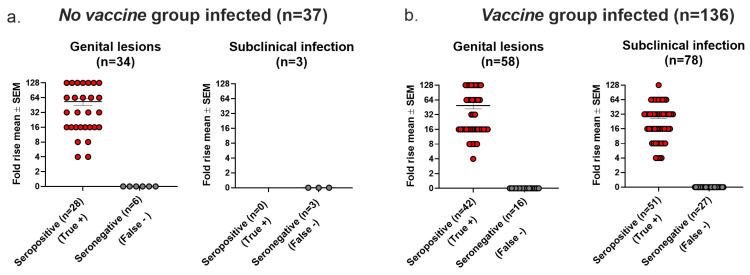
Seroconversion accuracy for confirming HSV-2 as cause of genital lesions and for detecting subclinical (latent) infection. (**a**) Seroconversion results in *No Vaccine* group. (**b**) Seroconversion results in *Vaccine* group.

Seroconversion is used in human vaccine trials to confirm HSV as the cause of genital lesions if PCR or virus cultures are negative or not performed and to diagnose latent infection in individuals without genital lesions [1,2,3]. For the analysis in this section, we defined infection using the Lesions/Latency criteria. In the *No vaccine* group, 34 animals had genital lesions, of which 6 (18%) were seronegative (false negative). All three animals that had subclinical (latent) infection without genital lesions were seronegative (false negative) (Figure 6a). In the *Vaccine* group, 16 of 58 (28%) animals with genital lesions were seronegative (false negative), and 27 of 78 (35%) animals with subclinical (latent) infection without genital lesions were seronegative (false negative) (Figure 6b). We conclude that seroconversion had high false negative rates when used to confirm genital lesions or to detect latent infection in the absence of genital lesions in the *No vaccine* and *Vaccine* groups.

### 5.2. Accuracy of Seroconversion Increases as Vaccine Efficacy Improves

We used three vaccine formulations: (i) the gD2 subunit protein; (ii) the gC2, gD2, and gE2 trivalent subunit proteins; and (iii) the gC2, gD2, and gE2 trivalent protein-modified mRNA-LNP. Using the Lesions/Latency criteria to define infection, the gD2 subunit vaccine was the least efficacious, with 80% of animals becoming infected compared to 66% in the trivalent protein group, and with 55% in the trivalent mRNA group (*p* = 0.0259 comparing gD2 with trivalent mRNA, two-tailed Fisher’s exact test) [12,18]. We assessed whether the accuracy of seroconversion varied depending on vaccine efficacy. Seroconversion accuracy was 52% in the gD2 vaccine group, 68% in the trivalent protein vaccine group, and 78% in the trivalent mRNA group (Table 5). We conclude that the accuracy of seroconversion is unacceptably low even for the best performing vaccine, the trivalent mRNA vaccine, which achieved sterilizing immunity (no genital lesions and no latent infection) in 62/137 (45%) animals (Table 5).

## 6. Discussion

The guinea pig prophylactic vaccine studies were performed over 10 years using three vaccine formulations, namely a vaccine involving the gD2 subunit protein with CpG and alum; trivalent gC2, gD2, and gE2 subunit proteins with CpG and alum; and trivalent gC2, gD2, and gE2 nucleoside-modified mRNA-LNP [12,17,18]. The advantages of the guinea pig model compared to human vaccine studies are that we can conduct challenge studies with HSV-2 in animals; we know the day the animal was inoculated and can collect vaginal swabs at predetermined times post inoculation; and we can harvest DRGs at the end of the study. These advantages improve our ability to determine whether an animal is infected. We also can collect sera at appropriate times to optimize our chances to detect seroconversion. Under these optimal conditions, we determined that seroconversion performed poorly as an indicator of infection in the *No vaccine* and *Vaccine* groups with overall accuracy ranging between 71% and 79% depending on the definition used. We obtained many false negative results in both vaccine groups and many false positive results in the *Vaccine* group. The false positive results indicate that seroconversion occurred in vaccinated animals that had no lesions and no latent infection (clinically insignificant infection) but not in unvaccinated animals. This level of accuracy in unvaccinated and vaccinated animals is very concerning for an assay that is used to judge vaccine efficacy.

In humans, gG ELISA on a single serum sample is inaccurate for distinguishing HSV-1 from HSV-2 infection particularly for samples with low positive results for HSV-2 antibodies [19]. The three manuscripts that reported results of large human efficacy trials each used seroconversion to help establish the diagnosis of infection [1,2,3]. Seroconversion was measured by Western blotting in the first trial that used gB2 and gD2 immunogens [1]. Twenty-eight subjects that seroconverted by Western blotting were also evaluated using a gG2 protein assay. The concordance between the two assays for seroconversion was 100%. The second manuscript contained two substudies that involved immunizing with a gD2 vaccine [2]. Western blotting was used to detect seroconversion in substudy 1 (double seronegative subjects), while HSV-1 gG (gG1) and gG2 ELISA were used in substudy 2 (subjects of any HSV serostatus). No comparisons were made between gG1/gG2 and Western blot assays in this manuscript. The third publication also used a gD2 vaccine [3]. HSV-1 gG1 and gG2 ELISA were used to detect seroconversion (HerpeSelect-2^TM^), while Western blotting was used to confirm all seroconversions, but no comments were provided about whether some results were discordant (supplementary appendix [3]). Although the information available is limited, the data support concordance between the two assays when paired sera are available. The poor performance of seroconversion for detecting infection in our study is unlikely to be related to our decision to use gG2 ELISA rather than Western blotting to measure seroconversion in most animals.

We noted that seroconversion accuracy improved as vaccine efficacy improved. The best performing immunogen was the trivalent mRNA-LNP vaccine, while the least effective was the gD2 protein vaccine. This observation suggests that seroconversion may have value in epidemiologic studies using paired sera to evaluate new (incident) infections if a highly effective vaccine is approved for general use.

An issue is whether other tests are available to replace seroconversion to confirm the diagnosis of genital lesions or detect latent infection in vaccine trials. For genital lesions, swab samples for HSV PCR can be collected by the subject at home to eliminate the need to travel to the clinic or risk that lesions will heal before the subject is seen in the clinic [3,23,24]. Home swabbing by subjects for HSV-2 DNA or other sexually transmitted infections is as accurate as when samples are collected by healthcare providers in the clinic [25,26]. To detect latent infection, subjects can be instructed on how to self-collect daily genital swabs at home over one or more months [23,24]. Comparing home swabbing with seroconversion as a confirmatory test for genital lesions or to detect HSV latency may enable us to replace seroconversion with home swabbing in prophylactic genital herpes vaccine trials.

## Figures and Tables

**Table 1:** Guinea pigs included in seroconversion study.

Conditions	*No Vaccine* (*n* = 43)	*Vaccine* (*n* = 224)
HSV-2 inoculum	4 × 10^5^ PFU	5 × 10^5^ PFU
Vaccine and dose	None (*n* = 43)	gD2 protein, 10 μg (*n* = 25) gC2/gD2/gE2 proteins, 10 μg each (*n* = 62) gC2/gD2/gE2 mRNA, 5, 10, or 20 μg each (*n* = 137)
Days scored for genital lesions	≥41	≥41
Days tested for vaginal shedding of HSV-2 DNA after day 28	≥20	≥20
Interval between acute and convalescent sera	2 months	2 months

**Table 2:** Positive indicators of infection in the *No vaccine* and *Vaccine* groups.

Indicator of Infection	*No Vaccine* (*n* = 43)	*Vaccine* (*n* = 224)
Genital lesions	34 (79%)	58 (26%)
Vaginal shedding HSV-2 DNA	30 (70%)	112 (50%)
HSV-2 DNA in DRG	24 (56%)	40 (18%)
Any positive assay (animal considered infected)	37 (86%)	136 (61%)
All assays negative (animal considered uninfected)	6 (14%)	88 (39%)

**Table 3:** No seroconversion or infection after inoculation of UV-inactivated virus.

Condition	*No Vaccine* (*n* = 8)	*Vaccine* (*n* = 10)
Peak vaginal titers on day 2 or day 4	Neg 8/8 animals	Neg 10/10 animals
Days with genital lesions	0/160 days	0/310 days
Days with vaginal shedding HSV-2 DNA	ND *	0/90 days
HSV-2 DNA in DRG	ND	0/10 animals
gG2 seroconversion	0/8 animals	0/10 animals

* ND, not done.

**Table 4:** Seroconversion using Lesions/Latency, Replication, or Combined criteria for infection.

Seroconversion	*No Vaccine*			*Vaccine*		
	Lesion/Lat. (*n* = 43)	Replication (*n* = 39)	Combined (*n* = 39)	Lesion/Lat. (*n* = 224)	Replication (*n* = 193)	Combined (*n* = 193)
False neg.	9/37 (24%)	8/33 (24%)	10/35 (29%)	43/136 (32%)	37/121 (31%)	52/141 (37%)
False pos.	0/6 (0%)	0/6 (0%)	0/4 (0%)	19/88 (22%)	9/72 (13%)	4/52 (8%)
Accuracy	34/43 (79%)	31/39 (79%)	29/39 (74%)	162/224 (72%)	147/193 (76%)	137/193 (71%)

According to the criteria used to define infection by Lesions/Latency, at least one of the following was required: genital lesions, vaginal shedding of HSV-2 DNA, or DRG positive for HSV-2 DNA. Criterion for Virus Replication was at least one day with a positive virus culture performed on days 2 and 4 post inoculation. Criterion for Combined method required any criteria noted for Lesions/Latency and Virus Replication. Lat., latency.

**Table 5:** Accuracy of seroconversion parallels vaccine efficacy.

Vaccine	Infected ^		Not Infected		Total Protected	Seroconversion Accuracy
	Seropos (True +)	Seroneg (False −)	Seropos (False +)	Seroneg (True −)		
gD2 (*n* = 25)	10	10	2	3	5/25 (20%)	13/25 (52%)
Tri-protein (*n* = 62)	26	15	5	16	21/62 (34%)	42/62 (68%)
Tri-mRNA (*n* = 137)	57	18	12	50	62/137 (45%)	107/137 (78%)

^ Infection was defined using the Lesions/Latency criteria requiring one or more of the following: genital lesions, vaginal shedding of HSV-2 DNA, or HSV-2 DNA in DRG.

## Data Availability

All the relevant data are included in the manuscript.
